# Supplementary material for: Effects of early life stress and subsequent re-exposure to stress on neuronal activity in the lateral habenula
Source: Neuropsychopharmacology. 2022 Nov 12;48(5):745–53. doi: 10.1038/s41386-022-01493-0 (PMC10066304; doi:10.1038/s41386-022-01493-0)
Supplement: Supplementary file 1 — Supplementary information [file 41386_2022_1493_MOESM1_ESM.pdf]

Supplementary information

Effects of early life stress and subsequent re-exposure to stress on neuronal activity in the lateral habenula

Jack F. Webster[1], Sanne Beerens[1] & Christian Wozny[1,2]

1 Strathclyde Institute for Pharmacy and Biomedical Sciences, University of Strathclyde, Glasgow, G4 0RE, United Kingdom

2 MSH Medical School Hamburg, Medical University, Institute for Molecular Medicine, 20457 Hamburg, Germany

For correspondence: christian.wozny@medicalschool-hamburg.de

Methods

Animals

All procedures were approved by the Ethics committee of the University of Strathclyde, Glasgow, and carried out in accordance with the relevant UK legislation (the Animals (Scientific Procedures) Act, 1986). Male and female mice from each strain were used in this work, and unless otherwise stated, data were pooled between genders. Mouse strains used in this study were C57BL/6J, SOM-IRES-Cre heterozygous mutants (Jax ID 018973; Taniguchi *et al.*, 2011), and their wild-type littermates. All animals were kept on a 12:12 light/dark cycle under standard group housing conditions with unlimited access to water and normal mouse chow, unless otherwise stated.

Maternal separation procedure

From the point of visually identified females were housed individually, and date of littering was counted as postnatal day 0 (P0). Litters of 5-10 pups were used for these procedures. At P6, the litter was divided into even (±1 pups) groups of MS and control (CTRL) pups. MS pups were then separated from the mother into individually isolated compartments in a heated cage in a separate room for 6 hours per day from P6-16, and then weaned early at P17. Separation started each day between 08:00 and 10:00. CTRL pups remained with the mother and were weaned at P21. At the early weaning age of the MS pups (P17), they are often unable to consume normal mouse chow, and as such we provided them with human baby food for the final 2 days of MS to allow them to habituate to this. This was then provided for several days post weaning for both MS and CTRL mice to allow them to rapidly develop to the point where they could be sustained on normal mouse chow. Following weaning, both groups were then group housed (2-5 mice per cage) and allowed to develop to adulthood when further testing commenced.

Behavioural testing and acute restraint procedure

Full details on behavioural testing and normalised behavioural Z score analysis are as follows:

Sucrose preference. Mice were first single housed, and then were given a choice between 2 bottles of water to habituate them to having 2 spouts in the cage. The following day (Day 1), both bottles were refilled and replaced. The left bottle again contained water, and the right bottle was filled with 1 % sucrose solution. On the third day (Day 2), bottles were refilled and replaced, this time with the 1 % sucrose solution bottle on the left, and water on the right. On the final day (Day 3), the positions of the bottles were pseudorandomised such that they alternated between cages. For all days, bottles were weighed before being added to the cage and again 24 hours later, and the consumption of fluid from each was used to calculate sucrose preference.

Open field test. Open field testing was carried out the day following the completion of sucrose preference testing. Mice were put in a white square arena (40 x 40 x 35 cm) within a room with standard lighting conditions, and allowed to freely explore for 5 minutes. Activity was recorded using an overhead camera, and videos were analysed using the analysis programme ToxTrac (Rodriguez *et al.*, 2018), with the outermost 5 cm of the arena being classed as the borders.

Splash test. Splash test was carried out on the same day as open field testing, at least 2 hours after completion of this test. This was carried out in the home cage. 30 minutes prior to testing, all environmental enrichment (nesting material and plastic house) was removed from the home cage. Mice were then sprayed once on the dorsal coat with a solution of 10 % sucrose. To avoid the scent of sucrose distracting the animals during testing, mice were sprayed outside of the home cage. The mouse was then immediately returned to the home cage, the lid replaced, and activity recorded for 5 minutes using an overhead camera. Analysis was performed using the analysis programme BORIS (Friard and Gamba, 2016).

Acute restraint. Mice were restrained within modified handling tubes for a period of 1 hour. Upon completion of the acute restraint procedure, mice were either immediately sacrificed by cervical dislocation for preparation of acute brain slices, or returned to the home cage for 1 hour before transcardial paraformaldehyde (PFA) perfusion to assess c-Fos expression. Mice that were to be sacrificed for brain slice preparation immediately after restraint remained in group-housing conditions. Mice that were to undergo PFA perfusion were single-housed for at least 2 days prior to restraint.

Behavioural Z scoring. Behavioural Z scores were calculated as previously described (Guilloux *et al.*, 2011). Briefly, a normalised Z score was calculated for each of the following 7 behavioural readouts: sucrose preference on days 1, 2 and 3; percentage time spent in borders in the open field test and; percentage time spent grooming, latency to first grooming bout and time rearing in the splash test. Behavioural Z scores were then calculated as the average of these 7 Z scores for each individual animal.

Stereotaxic viral injections

SOM-IRES-Cre heterozygous mice (approximately 8-9 weeks old) were deeply anaesthetized via inhaled isoflurane (5% for induction; 1–2% for maintenance), transferred to a stereotaxic frame (Narishige, Tokyo, Japan) and were subcutaneously injected with the analgesics carprofen (5 mg/kg) in the nape and lidocaine (4 mg/kg) under the scalp. Intracranial injections were made using a glass micropipette pulled using a PC-100 vertical puller (Narishige, Tokyo, Japan). Under aseptic conditions, the skull was exposed and a small burr hole was drilled bilaterally above the basal forebrain (BF). Stereotaxic coordinates (from Bregma) were as follows: AP 0.45; ±1.3; depth 5.8. The injection capillary was then advanced and viral vector solutions were

injected at a rate of 100 nL/min using a pressure microinjector (Narishige, Tokyo, Japan). Viral vector solutions used in this study were AAV9-EF1a-DIO-hChR2(H134R)-EYFP, titre 1.8x10^{13} vg/mL, 200 nL; AAV9-pCAG-FLEX-EGFP-WPRE, titre 2.5x10^{13} vg/mL, 100 nL injected (both from Addgene, Massachusetts, US). Following injection, the needle was left for at least 10 minutes to allow the virus to diffuse before being slowly withdrawn. Animals were allowed to recover from anaesthesia on a heat pad. Following completion of surgery, animals were given at least two weeks to allow expression of the virus before acute slice preparation for electrophysiology. Assessment of viral spread was carried out using either a fluorescent camera (Olympus XM10; Olympus, Southend-on-Sea, UK) with a 4X objective, or a Leica SP8 confocal microscope.

Acute brain slice preparation

Mice were humanely euthanized by cervical dislocation and immediately decapitated, and brains were rapidly removed and transferred to ice-cold oxygenated (95% O2; 5% CO2) sucrose-based artificial cerebrospinal fluid (ACSF) solution containing (in mM): sucrose 50, NaCl 87, NaHCO3 25, KCl 3, NaH2PO4 1.25, CaCl2 0.5, MgCl2 3, sodium pyruvate 3 and glucose 10. Brains sections containing the lateral habenula were then cut in the coronal plane at 250 µm where in vitro optogenetic experiments were to be performed, or 300 µm for all other slice experiments, on a Leica VT1200S vibratome (Leica Biosystems, Newcastle-upon-Tyne, UK). Following sectioning, slices were incubated in oxygenated sucrose-based ACSF at 35 °C for 30 minutes, and then incubated for a further 30 minutes at room temperature in ACSF containing (in mM) NaCl 115, NaHCO3 25, KCl 3, NaH2PO4 1.25, CaCl2 2, MgCl2 1, sodium pyruvate 3 and glucose 10. Following the incubation period, slices were stored at room temperature in oxygenated ACSF until required for electrophysiological recordings.

In vitro electrophysiological recordings

Individual slices were transferred to a recording chamber and continually perfused with oxygenated ACSF at a flow rate of 2–3 mL/min and visualized with a Luigs and Neumann LN-Scope System (Luigs and Neumann, Ratingen, Germany). Neurons suitable for whole-cell recordings were identified under a 60X objective. For transgenic animals which expressed a fluorescent reporter protein (eGFP or eYFP), fluorescent reporters were excited using a LED coupled through the 60X objective (pE-300ultra, Cool LED, Andover, UK), and reporter-expressing somata or terminal fields were visualized with an Olympus XM10 fluorescent camera (Olympus, Southend-on-Sea, UK)). Recordings were made with a Multiclamp 700B Amplifier (Molecular Devices, California, USA). For current clamp recordings, and for recording optogenetically-driven postsynaptic currents, glass micropipettes were filled with a solution containing (in mM) potassium gluconate 125, Hepes 10, KCl 6, EGTA 0.2, $MgCl_2$ 2, Na-ATP 2, Na-GTP 0.5, sodium phosphocreatine 5, and with 0.2% biocytin, and pH was adjusted

to 7.2 with KOH. For spontaneous postsynaptic current measurement experiments, a potassium chloride-based intracellular solution was used consisting of (in mM) potassium chloride 145, EGTA 0.1, Hepes 10, NaATP 2 and $MgCl_2$ 2, pH adjusted to 7.2. To pharmacologically isolate inhibitory currents, these experiments were performed in the presence of AMPA and NMDA receptor blockade (10 µM NBQX and 50 µM D-AP5 respectively; Tocris, Bristol, UK). To isolate excitatory currents, experiments were performed in the presence of $GABA_A$ and $GABA_B$ blockade (5 µM SR-95531 and 10 µM CGP-52432 respectively; Tocris, Bristol, UK).

For current clamp recordings, a gigaseal was first achieved in voltage-clamp configuration when the pipette was in the immediate vicinity of the neuron. The neuron was then held at a potential of -60 mV, and whole-cell configuration was achieved by rupturing the membrane with a series of negative pressure pulses. Once in whole-cell patch mode, the intrinsic properties of LHb neurons were assessed by switching to current-clamp configuration (*I = 0)* and recording spontaneous activity for a period of 2-3 minutes. Neurons which fired action potentials with a frequency of at least 0.5 Hz were classed as spontaneously active. Spontaneously active neurons were further sub-classified as bursting based on the presence of a highly distinctive and rhythmic 'calcium plateau' (Yang *et al.*, 2018) from which a burst of action potentials arose, and a hyperpolarized resting membrane potential of less than -58 mV. Spontaneously active neurons which did not meet these criteria were classed as tonically active. Following spontaneous activity recording, current-spike input-output relationships were tested by injecting sufficient holding current to hold the neuron at a potential of -55 mV, and a series of depolarising current steps were injected (0-100 pA; 10 pA steps). Holding current was then removed, and a second series of current steps were injected (-50-100 pA; 10 pA steps) to assess both rebound bursting properties, and spiking properties at rest.

For optogenetically-driven postsynaptic inhibitory current recordings, whole-cell configuration was achieved as above, and recordings were performed in voltage-clamp configuration at a holding potential of -50 mV. During recording, a single blue LED pulse (1 ms) of increasing intensity (1-50%; 3-4 trials per intensity) was applied to induce a postsynaptic current, with the amplitude being measured as an average of 3 trials. Upon completion of these experiments, the same neurons were then held in current clamp configuration to assess both physiological properties, and the capacity for optogenetically-driven inhibitory synaptic transmission to induce rebound bursting.

For spontaneous current measurements, whole-cell configuration was achieved at -70 mV, and spontaneous synaptic activity was recorded for a period of 2 minutes. For these experiments, recordings were completed no more than 4 minutes after break-in, as we observed rapid reduction in synaptic event frequency using the aforementioned high chloride intracellular solution.

Series resistance was monitored throughout. All neuronal voltage and current signals were low pass-filtered between 2 and 10 kHz and acquired between 10 and

25 kHz using an ITC-18 digitizer interface (HEKA, Pfalz, Germany). The data acquisition software used was Axograph X.

Transcardial perfusion and sectioning

Mice were terminally anaesthetized by intraperitoneal injection with an overdose cocktail of 50% lidocaine and 50% pentobarbital. Once anaesthetized sufficiently to be non-responsive to tail and toe pinch stimuli, mice were perfused through the left ventricle with 0.1 M PBS followed by perfusion with 4% PFA dissolved in PBS. Brains were then removed and fixed overnight in 4% PFA in PBS, after which they were cryoprotected in a solution containing 30% sucrose in PBS. Brains were left in this solution until they dropped to the bottom of the tube, at which point the 30% sucrose solution in PBS was replaced with fresh 30% sucrose solution. Once the brain dropped for a second time, it was considered ready for sectioning. This was performed by embedding in OCT compound (VWR, Leicestershire, UK) and freezing with a dry ice bath. Once frozen, brains were sectioned on a Leica SM2010 R microtome (Leica Biosystems, Newcastle-upon-Tyne, UK) at 50 - 60 µm.

Immunohistochemistry and confocal imaging

Following sectioning, slices were washed 3 times in 0.1 M PBS, and then incubated for 30 minutes in a blocking solution consisting of 5% normal goat serum (NGS) and 0.3% Triton X-100. Blocking solutions was then removed, and slices were incubated on a shaker at room temperature overnight in a primary antibody mixture containing 0.3% Triton in PBS and rabbit anti-c-Fos (1/10000; ab190289; Abcam, Cambridge, UK). Upon completion of the primary incubation step, slices were washed 2 × 5 minutes in 0.1 M PBS and incubated for 3 hours in a solution containing donkey anti-rabbit conjugated to Alexa Fluor 647 (1/500 dilution; Invitrogen, UK). After secondary antibody incubation, slices were washed for 3 times in 0.1 M PBS and mounted on glass slides using Vectashield medium containing DAPI (Vector Labs, Peterborough, UK).

Slices were imaged on a Leica SP8 confocal microscope using a 20X objective at 3 different points from Bregma along the rostrocaudal axis, spaced approximately 300 µm apart. For these experiments, imaging was performed with a 633 nm laser at 1 % max intensity, 2 µm z-steps.

Statistical analysis

Statistical analysis was carried out in GraphPad Prism 9.3.1. For pairwise comparisons, an unpaired T test was used where at least one data set was found to be normally distributed (tested with a Shapiro-Wilk normality test). Where both sets of data failed a normality test, a Mann-Whitney test was used. For grouped data, 2-way

ANOVA analysis was performed, and data were considered statistically significant when a p value of < 0.05 was found between the column factor (ie. between CTRL and MS mice).

References

Friard, O. & Gamba, M. BORIS: a free, versatile open-source event-logging software for video/audio coding and live observations. *Methods Ecol. Evol*. **7**, 1325–1330 (2016).

Guilloux, J. P., Seney, M., Edgar, N. & Sibille, E. Integrated behavioral z-scoring increases the sensitivity and reliability of behavioral phenotyping in mice: Relevance to emotionality and sex. *J. Neurosci. Methods* **197**, 21–31 (2011).

Rodriguez, A., Zhang, H., Klaminder, J., Brodin, T., Andersson, P. L. & Andersson, M. ToxTrac: A fast and robust software for tracking organisms. *Methods Ecol. Evol.* **9**, 460–464 (2018).

Taniguchi, H., He, M., Wu, P., Kim, S., Paik, R., Sugino, K., Kvitsani, D., Fu, Y., Lu, J., Lin, Y., Miyoshi, G., Shima, Y., Fishell, G., Nelson, S. B. & Huang, Z. J. A Resource of Cre Driver Lines for Genetic Targeting of GABAergic Neurons in Cerebral Cortex. *Neuron*, **71**, 995–1013 (2011).

Yang, Y., Cui, Y., Sang, K., Dong, Y., Ni, Z., Ma, S. & Hu, H. Ketamine blocks bursting in the lateral habenula to rapidly relieve depression. *Nature* **554**, 317–322 (2018).

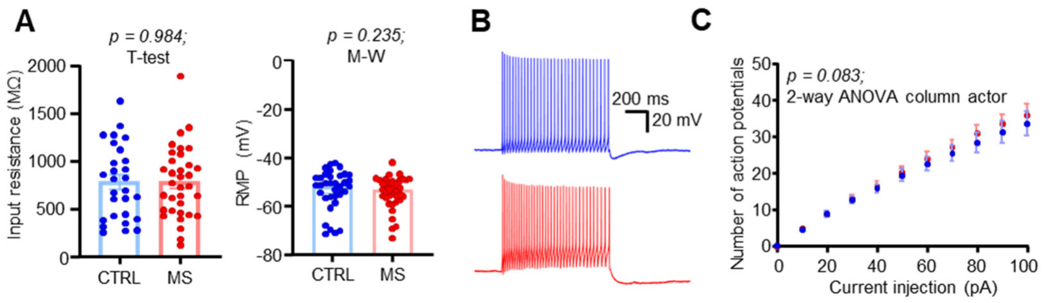

Supplementary figure 1: Effects of MS on passive physiological properties and intrinsic excitability.

(A) Comparison plots of passive physiological properties between conditions. (B) Example traces of action potential discharge in response to a 100 pA current step in both conditions. (C) Input-output plot of input current against mean number of induced action potentials.

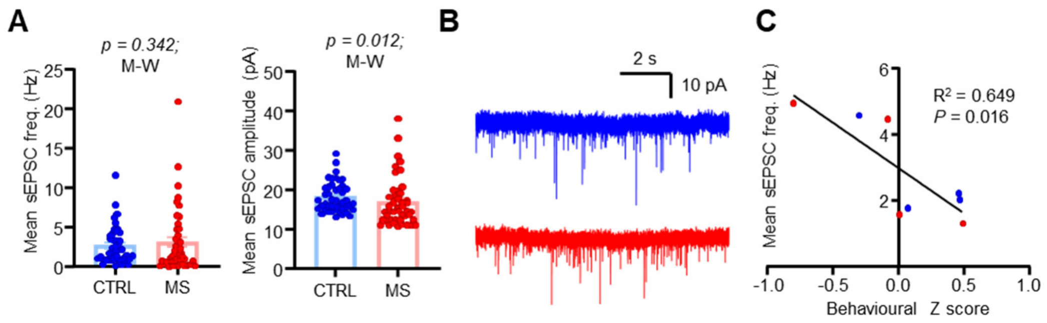

Supplementary figure 2: Effects of MS on spontaneous excitatory input to the LHb.

(A) Comparison plots of sEPSC frequency and amplitude between conditions. (B) Example sEPSC recordings in both conditions. (C) XY plot of behavioural z score against mean sEPSC frequency calculated for each individual mouse recorded from. Mean sEPSC scores are calculated as the mean sEPSC frequency of all cells recorded from each mouse.

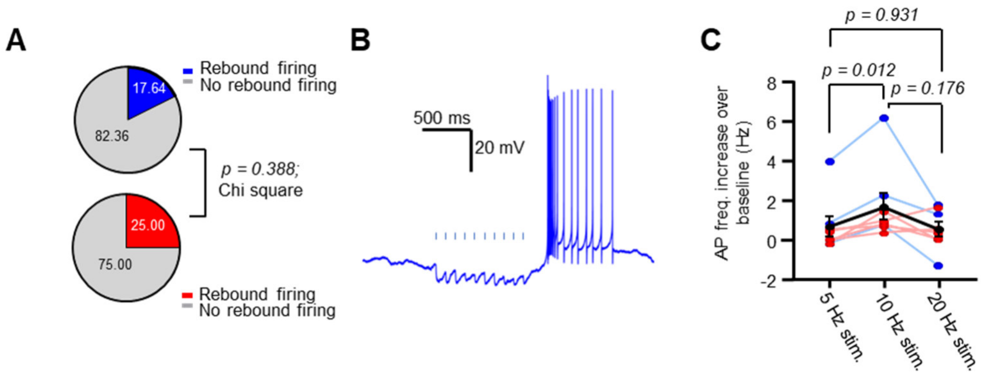

Supplementary figure 3. Optogenetic stimulation of inhibitory forebrain terminals can drive rebound firing within the LHb.

(A) Percentages of neurons which exhibited rebound firing following optogenetic stimulation in both conditions. (B) Example trace from a neuron recorded in a CTRL mouse. (C) Comparison of rebound firing between stimulation frequencies as an increase over baseline spontaneous firing. Note here that as no difference was observed between CTRL and MS mice, these are displayed on the same plot. *p* values here are from Tukey's multiple comparisons test.

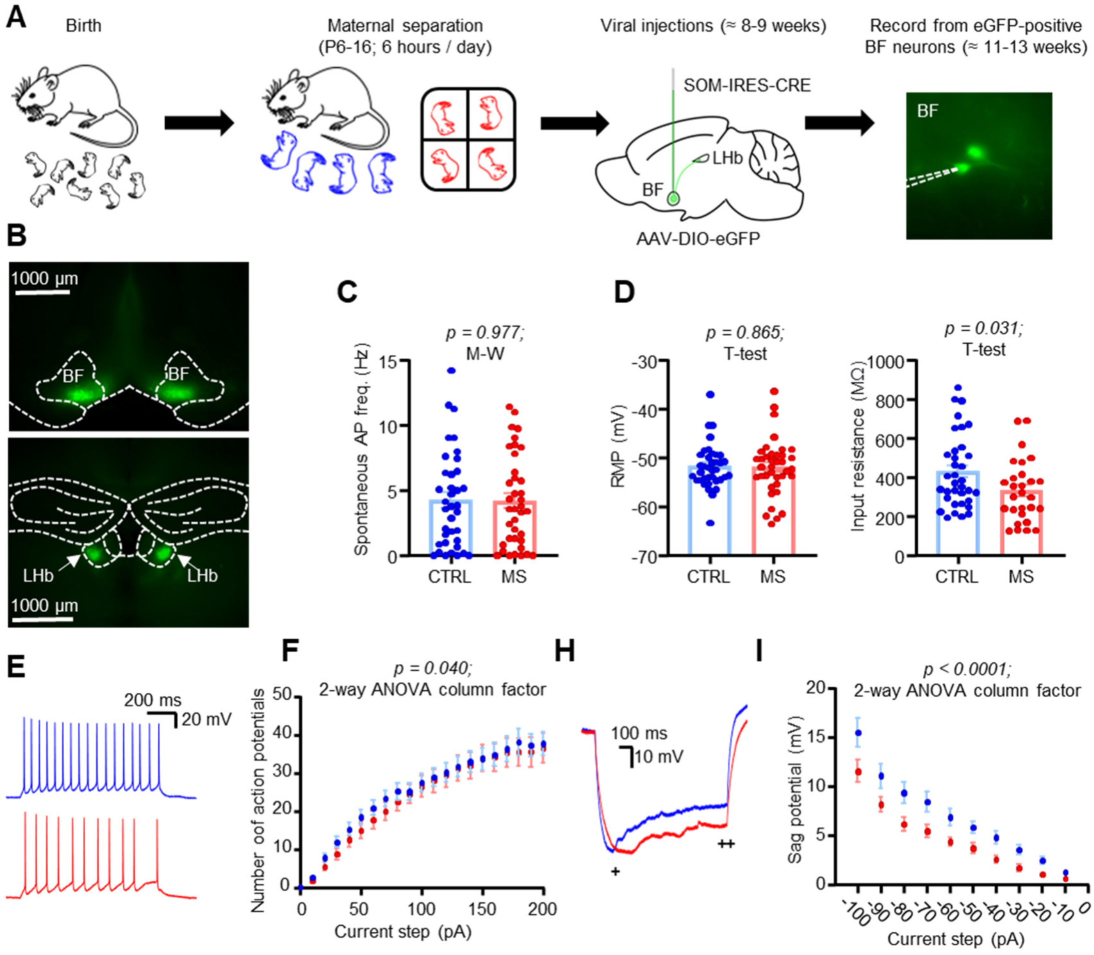

Supplementary figure 4: MS alters physiological properties of putative presynaptic neurons within the basal forebrain

(A) Schematic illustrating experimental timeline for recording from putative presynaptic basal forebrain neurons. (B) Example images of infected eGFP-positive neurons at injection site (top) and of terminal fields in the LHb (bottom) following viral injection. (C) Comparison plots of mean spontaneous activity frequency and of (D) passive physiological properties between neurons. (E) Example traces and (F) Input output plot of induced action potentials in response to depolarising current steps for both conditions. (I) Example traces and (J) plot of input current against sag potential in both conditions. Sag potential was calculated as the difference of the peak (+) and the steady state (++) of the membrane hyperpolarisation induced in response to hyperpolarising current steps.

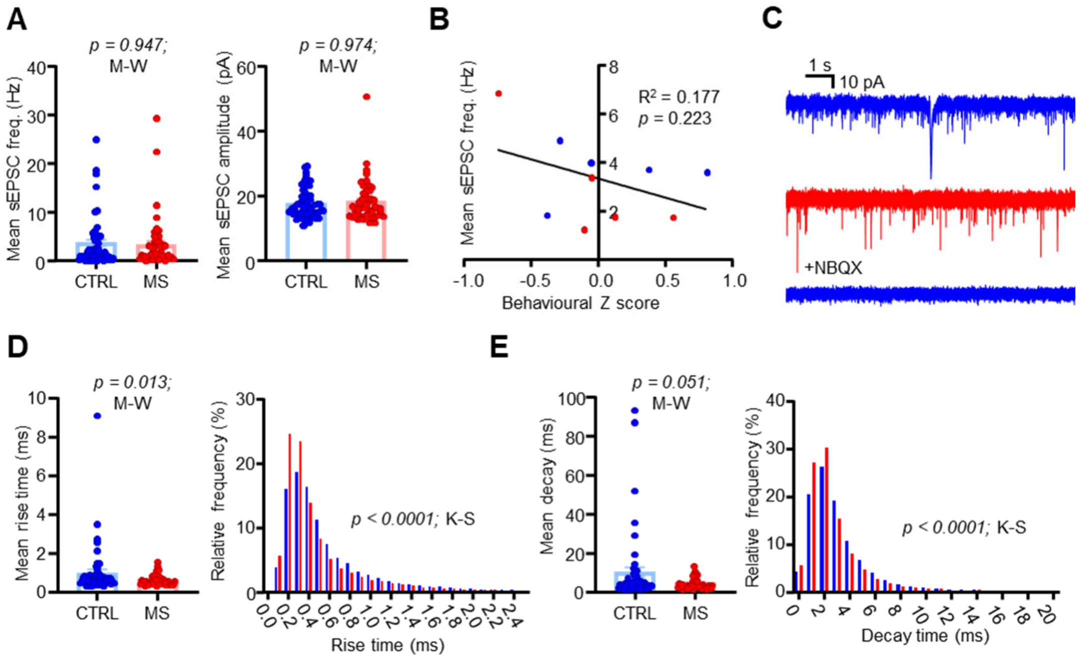

Supplementary figure 5: MS alters AMPA receptor signalling in response to acute stress within the LHb.

(A) Comparison plots of sEPSC frequency and amplitude between conditions. (B) XY plot of behavioural z score against mean sEPSC frequency calculated for each individual mouse recorded from. Mean sEPSC scores are calculated as the mean sEPSC frequency of all cells recorded from each mouse. (C) Example sEPSC recordings from neurons from CTRL (top) and MS (mid) mice, and from a CTRL mouse in the presence of 10 μM NBQX (bottom). (D) Left: comparison plots of sEPSC rise time between conditions. Probability distribution histogram comparing mean rise time distribution for all recorded neurons between conditions. Data are 0.1 ms bins. (E) As for D, with decay time. For probability distribution histogram, data are 1 ms bins.

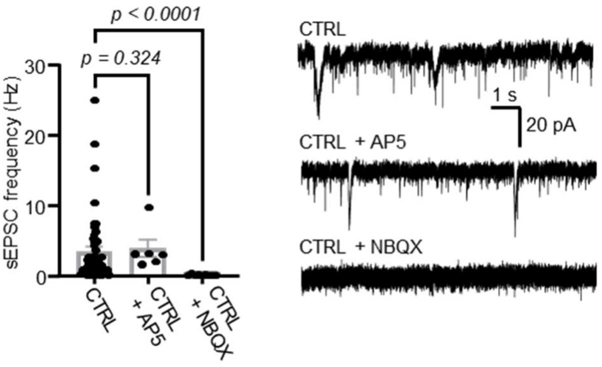

Supplementary figure 6: Spontaneous excitatory currents within the LHb are AMPA-mediated.

Left: comparison plot of sEPSC frequencies recorded in cells without AMPA or NMDA antagonization, with 50 µM AP5 and with 10 µM NBQX. Right: example recordings from three different neurons from the same animal for each of the conditions shown in the plot on the left.
